# An Emotion Regulation and Impulse Control (ERIC) Intervention for Vulnerable Young People: A Multi-Sectoral Pilot Study

**DOI:** 10.3389/fpsyg.2021.554100

**Published:** 2021-04-01

**Authors:** Kate Hall, George Youssef, Angela Simpson, Elise Sloan, Liam Graeme, Natasha Perry, Richard Moulding, Amanda L. Baker, Alison K. Beck, Petra K. Staiger

**Affiliations:** ^1^School of Psychology, Deakin University, Geelong, VIC, Australia; ^2^Centre of Drug Use, Addictive and Antisocial Behavior Research, Deakin University, Geelong, VIC, Australia; ^3^Centre for Adolescent Health, Murdoch Children's Research Institute, Melbourne, VIC, Australia; ^4^School of Medicine and Public Health (Psychiatry), University of Newcastle, Newcastle, NSW, Australia

**Keywords:** emotion regulation, comorbidity, adolescence, young adults, treatment

## Abstract

**Objective:** There is a demonstrated link between the mental health and substance use comorbidities experienced by young adults, however the vast majority of psychological interventions are disorder specific. Novel psychological approaches that adequately acknowledge the psychosocial complexity and transdiagnostic needs of vulnerable young people are urgently needed. A modular skills-based program for emotion regulation and impulse control (ERIC) addresses this gap. The current one armed open trial was designed to evaluate the impact that 12 weeks exposure to ERIC alongside usual care had on young people's ability to regulate emotions, as well as examine potential moderating mechanisms.

**Methods:** Seventy nine young people (50.6% male; *M* = 19.30; *SD* = 2.94) were enrolled to the 12 week intervention period. Twenty one practitioners from youth and community health services delivered relevant ERIC modules adjunct to usual care. Linear mixed effects regression (with random intercept) was used to examine change over time across the primary outcome of emotion dysregulation and secondary outcomes of depression, anxiety, stress, experiential avoidance and mindfulness. Moderation analyses were conducted to test whether the magnitude of change in emotion dysregulation moderated change over time in secondary outcomes.

**Results:** Analyses revealed significant improvement in the primary outcome of emotion dysregulation with a moderate effect size (Mean Change = −10.24, 95% CI (−14.41, −6.06; Cohen's d_av_ = −0.53), in addition to decreases in the secondary outcomes of depression, anxiety, stress and experiential avoidance. No improvements in mindfulness were reported. Moderation analyses revealed that the residualised change over time in emotion dysregulation moderated the change over time in symptoms of distress, depression, anxiety, stress, experiential avoidance, and mindfulness.

**Conclusion:** Reductions in the severity of emotion dysregulation, depression, anxiety, stress and experiential avoidance are promising, and were evident despite the complexity of the participants and the diversity of the service setting. The improvements found in each outcome were only observed for those young people whose emotion regulation also improved, providing preliminary evidence for the role of emotion regulation as a key treatment target in this population.

## Background

### Vulnerable Young People

Adolescents and young adults (aged 16–25; herein young people), accessing primary mental health, alcohol and other drug (AOD), youth justice, and community health services, are arguably one of the most vulnerable groups in society (Mitchell et al., 2016; Howe et al., 2017). These young people commonly have histories of social disadvantage and trauma, with the lifetime prevalence of abuse, neglect, child protection, criminal justice involvement, family conflict and homelessness exceptionally high (Lubman et al., 2016; Mitchell et al., 2016). Concurrent and interrelated mental health and substance use comorbidities are common, and pose ongoing risks to individuals' mental health and well-being (Couwenbergh et al., 2006; Davis and Kelly, 2012; Mitchell et al., 2016). However, despite the known morbidity and mortality associated with comorbid mental health and substance use issues [(Australian Institute of Health Welfare, 2018)] inadequate quality of care across these service systems is commonplace (McGorry, 2007). Referred to as the “quality chasm” (Institute of Medicine, 2006), a broad range of evidence-based psychosocial interventions are not being routinely delivered (Bearman and Weisz, 2015; Weisz, 2015).

There are multiple implementation barriers to the timely adoption of evidence-based psychological interventions (e.g., practitioner attitudes, organizational and resource factors, inadequate training, and intervention characteristics; see Mitchell, 2011 for a review). “Client characteristics” are also a significant barrier to implementing traditional evidence-based approaches. For example, Weisz (2015) observed that young people who seek treatment in youth services have greater diagnostic heterogeneity and psychosocial complexity than do young people participating in clinical trials, raising serious questions about the real-world clinical utility of evidence-based psychological approaches for young people. Indeed, a recent meta-analysis of five decades of youth psychological therapy found that empirical studies of treatment for concurrent disorders was limited (only 10 of 447 included trials), and produced strikingly smaller effect sizes than did single disorder treatment studies, with the impact of treatment on concurrent comorbidity not significant at post treatment follow up (Weisz et al., 2017). This in-depth appraisal highlights that treatment studies for concurrent conditions are in their infancy; indeed, participants with coexisting diagnoses are commonly excluded from trials (Weisz et al., 2017). This contrasts with service delivery settings, where: (a) concurrent diagnoses in young people are pervasive and may well be “the norm” (Angold et al., 1999); (b) certain disorders cluster together reliably (e.g., anxiety and depression; Brown and Barlow, 2009); and (c) diagnostic stability is low, with continuity from one disorder to another common (e.g., continuity from depression to anxiety and vice versa; Costello et al., 2003). Therefore, a critical question is whether our evidence-based protocols are applicable to vulnerable young people, given they commonly target single diagnoses or specific combinations of two coexisting disorders (Saunders and Kim, 2013). Evidently, novel psychological approaches are needed that not only adequately acknowledge the psychosocial complexity and transdiagnostic needs of vulnerable young service users but that can also be readily applied within real world clinical settings.

### Transdiagnostic Approaches

Transdiagnostic models of psychopathology consider the fundamental processes underlying multiple disorders and contribute to an explanatory framework for the common comorbidities between clusters of disorders, the diagnostic instability within individuals, and the acknowledgment that many dysfunctional processes are shared across disorders (see Mansell et al., 2009; Nolen-Hoeksema and Watkins, 2011, for reviews). Transdiagnostic approaches, therefore, offer a pragmatic and parsimonious response to complexity and facilitate the translation of evidence-based protocols into practice (Barlow et al., 2004; Brown and Barlow, 2009; Mitchell, 2011; Bearman and Weisz, 2015; Norton and Paulus, 2015; Marchette and Weisz, 2017). Transdiagnostic cognitive and behavioral treatments were first proposed over a decade ago (Fairburn et al., 2003) and are based on the premise that emotional and behavioral difficulties result from the dysfunction of relatively few core underlying processes, as many disorders share cognitive, emotional, behavioral and interpersonal features (McEvoy et al., 2009; Barlow et al., 2013). Consequently, transdiagnostic treatments aim to simultaneously intervene in multiple disorders by targeting aetiological factors or maintaining processes that are shared across disorders, within a unified framework (Harvey, 2013). Transdiagnostic approaches have subsequently been endorsed by paradigms that propose dimensional approaches that cut across diagnostic criteria such as the Research Domain Criteria (RDoC) and the clinical staging model of psychiatry (Scott et al., 2013; Cuthbert, 2014). A transdiagnostic approach to intervention development for vulnerable young people would therefore simultaneously address processes implicated as aetiological or maintaining factors present in clusters of disorders that commonly co-occur in young people, such as substance use disorders (SUD), disordered eating, borderline personality disorder (BPD) and self-harm (Sloan et al., 2019).

The list of transdiagnostic processes for psychopathology is extensive and includes both environmental (e.g., early childhood adversity and trauma) and intrapersonal risk factors (e.g., neuroticism and negative affectivity; see Nolen-Hoeksema and Watkins, 2011 for review). Of the intrapersonal transdiagnostic processes related to psychopathology, several cognitive processes (e.g., selective attentional biases; rumination; suppression; over-general memory; explicit selective memory; selective attention; attentional avoidance; interpretation and expectancy biases) and behavioral tendencies (e.g., avoidance, sleep disruption) have given rise to transdiagnostic cognitive behavioral therapy (CBT) approaches, and these have a growing evidence base (Mansell et al., 2009). Although transdiagnostic paradigms have their detractors (Fusar-Poli et al., 2019), and there have been mixed results for the comparable efficacy of these approaches to disorder specific approaches in anxiety and depressive disorders (Newby et al., 2015), the pragmatism and potential clinical utility of transdiagnostic approaches in youth services means that they have the potential to have an enormous impact on public health service provision for vulnerable young people.

### Deficits in Emotion Regulation

A sound transdiagnostic approach for vulnerable young people would be grounded in a unifying theoretical model that identifies or implicates common mechanisms across the cluster of mental health and substance use disorders commonly manifested in this cohort. Emotion regulation is one such potential transdiagnostic treatment target (Sloan et al., 2017). Deficits in emotion regulation have been implicated in the etiology and maintenance of anxiety, depression, disordered eating, BPD and SUD (Aldao et al., 2010; Gratz et al., 2015; Sloan et al., 2017, 2019), as well as deliberate self-harm and suicidal gestures (Kranzler et al., 2016), risky sexual behavior (Weiss et al., 2015), and aggressive behavior (Velotti et al., 2016). Further, childhood neglect, abuse and trauma, all of which are proposed to negatively impact the foundational relational processes that foster healthy emotion regulation during early childhood development (Cole et al., 2009; Crowell et al., 2009; Weiss et al., 2013b), are disproportionately evident in vulnerable young people (Mitchell et al., 2016). Together, this literature lends support to the relevance of emotion regulation as a potential transdiagnostic treatment target.

Emotion regulation is a multifaceted construct that can be broadly defined as the processes involved in modulating and managing the experience and expression of emotions (Gross, 1998). There are numerous and varied definitions of emotion regulation and conceptual ambiguity of this construct continues to exist (see Berking and Wupperman, 2012). However, the multidimensional model of emotion dysregulation proposed by Gratz and Roemer (2004) has been widely adopted in the clinical literature in relation to its association with numerous psychological disorders. Consequently, this model has clinical utility and underpins the intervention piloted in the present study. According to this model, emotion regulation encompasses the: (a) awareness and understanding of emotions; (b) acceptance of emotions; (c) ability to control impulsive behaviors and behave in accordance with desired goals when experiencing negative emotions; and (d) ability to use situationally appropriate emotion regulation strategies flexibly to modulate emotional responses as desired in order to meet individual goals and situational demands (Gratz and Roemer, 2004, p. 42–43). The relative absence of any or all of these abilities indicates the presence of emotion dysregulation.

Recent evidence suggests emotion dysregulation is amenable to change with cognitive and behavioral interventions, and that this change is associated with reduced symptom severity across the disorders most relevant to vulnerable young people. In a recent systematic review of 67 studies examining the treatment of anxiety, depression, SUD, disordered eating and BPD, engagement in maladaptive emotion regulation strategies and/or overall emotion dysregulation significantly decreased in all but three studies following a range of cognitive and behavioral psychological interventions (Sloan et al., 2017). Furthermore, concomitant decreases in symptom severity were observed across all classes of psychopathology (Sloan et al., 2017). This review not only highlighted that deficits in emotion regulation are responsive to CBT-based interventions, but also suggests that these changes may improve symptoms for the disorders relevant to vulnerable young people. In summary, converging lines of evidence highlight that emotion regulation skill development may be an important transdiagnostic target when working with young people who access services in youth justice, AOD and primary mental health.

### The ERIC Program

If we are to improve the social and emotional outcomes of vulnerable young people, intervention development needs to address known implementation barriers (i.e., guided by principles of co-design and participatory research), and extensive piloting and feasibility evaluation needs to occur within real world settings to address clinical validity (Craig et al., 2013). ERIC is an emotion regulation and impulse control modular CBT skills-based program, which was developed through a 3-year participatory research program in partnership with vulnerable young people and youth services across Australia (Hall and Simpson, 2018). ERIC was initially piloted via a case series with young people with multiple and complex mental health, substance use and psychosocial needs in a residential AOD treatment setting (Sloan et al., 2018). ERIC promotes sustained practice, coaching and intentional emotion regulation skill building and was designed to be delivered as an adjunct to existing service models across sectors that work with vulnerable young people.

ERIC is designed in a modular fashion to aid integration with the diverse psychosocial interventions commonly delivered by these sectors (e.g., life skills training, restorative justice, anger management, offending behavior programs, case management, youth outreach support, AOD and primary mental health). The modular framework permits clinicians to deliver the intervention flexibly in accordance with the emotion regulation needs of their specific clients (Chorpita et al., 2005). ERIC organizes the emotion regulation exercises, skills and processes into eight discrete domains: (1) reducing vulnerability; (2) emotional literacy; (3) flexible thinking; (4) allowing; (5) micro mindfulness; (6) tolerating discomfort; (7) decision making; (8) identity and values. Each of these domains has three desired emotion regulation outcomes that represent healthy development (See Table 1). The eight domains are informed by Gratz and Roemer's (2004) model and target important processes or strategies to help young people regulate their emotions and control impulsive behaviors linked to negative emotion (i.e., urgency (Cyders and Smith, 2008; Weiss et al., 2013a). There is an additional focus on helping young people reduce the valence of emotion in a timely way after they become distressed (i.e., self-soothing, Linehan, 1993).

**Table 1:** Content overview of the ERIC domains, worksheets and exercises delivered during the intervention.

**Dimension of ER, Gratz and Roemer, 2004**	**ERIC Domain**	**Worksheets**	**Exercise description**
The ability to use situationally appropriate emotion regulation strategies flexibly to modulate emotional responses in order to meet situational demands. Focuses on reducing reliance on maladaptive strategies: avoidance, suppression and rumination. Includes reducing valence of emotions through soothing.	Reducing vulnerability: (a) reduce rumination and suppression; (b) face up to avoidance; (c) practice good self-care habits	Interrupt rumination with 5-4-3-2-1	Psychoeducation on rumination and the relationship between rumination and emotion dysregulation^a^; exercise involving shifting attention through a counting task and physical grounding technique to interrupt rumination.
		Five self-care habits	Psychoeducation about calming down when distressed^d^; developing a personalized self-care plan with node-link map; prompts in the nodes to include reaching out to others, exercising and sleeping well, being mindful, eating well and compassion.
		Allowing pain… a little bit at a time	Psychoeducation on suppression and consequences of suppression for emotion regulation; exercise with visual prompt of pink elephant holding a banana with experiential exercise of trying not to think of the elephant for 30 s^e^^.^
		Facing up to avoidance	Psychoeducation on the impact of avoidance as an emotion regulation strategy; coping statements; a node-link map to plan a behavioral experiment^f^ to engage in graded exposure to an emotionally eliciting situation that has been avoided in the past.
The awareness and understanding of emotions: emotional clarity.	Emotional Literacy: (a) identify emotions and recognize their purpose; (b) identify how emotions impact thoughts, behaviors and body signals; (c) recognize the difference between helpful and unhelpful responses to emotions	Why should I regulate my emotions?	Psychoeducation regarding consequences of emotion dysregulation; insight building through identification of current strategies used to regulate emotions through checklist; identification of two emotion regulation habits to target in a change plan.
		Dissecting your feelings: 5 parts to strong emotions	Psychoeducation and a functional analysis^b^ of a recent situation to identify physical sensations, emotions, urges, cognitions and behaviors through a node-link map.
		Emotions are or mind's inbuilt alarm system	Psychoeducation regarding the evolutionary function of emotions^c^;
Ability to use situationally appropriate emotion regulation strategies flexibly to modulate emotional responses as desired in order to meet individual goals and situational demands.	Flexible Thinking: (a) to be able to look at a situation from another person's perspective; (b) to be aware of bias when interpreting a situation; (c) to accept other people's point of view as valid.	Put yourself in someone else's shoes	Psychoeducation on the cognitive model and an introduction of cognitive reappraisal^g^ as a helpful emotion regulation strategy; exercise that uses an ambiguous figure of two young people embracing, and four stories are generated to introduce different perspectives of the same situation.
Acceptance and tolerance of emotions.	Allowing: (a) to accept yourself and others; (b) to observe your thoughts and emotions without trying to change them; (c) to be kind and compassionate to ourselves	Allow space for all your feelings	Psychoeducation on acceptance of emotions as an adaptive emotion regulation strategy through an analogy of surfing; an exercise with emotional prompts to identify emotions that are currently avoided.
Acceptance and tolerance of emotions.	Micro Mindfulness: (a) to tune in to your mind and body; (b) to remain present in each moment; (c) to focus your attention.	Mindful breathing	Psychoeducation on mindfulness as an adaptive emotion regulation strategy; an analogy of spotlight of attention to aid mindful breathing; a step-by-step prompt for a 3 min breathing exercise^h^.
		Mindful lean	Psychoeducation on mindfulness^i^ as an adaptive emotion regulation strategy; a 3 min exercise focusing on the feet and toes when leaning forward to help check in with the present moment.
Ability to use situationally appropriate emotion regulation strategies flexibly to modulate emotional responses as desired in order to meet individual goals and situational demands	Tolerating Discomfort: (a) to sit with uncomfortable thoughts, feelings and body signals; (b) to resist an urge to engage in unhelpful behaviors; (c) to use distraction and self-comfort strategies to get through difficult situations	Shake off feelings	Psychoeducation on negative cognitive and attentional biases; node-link map to develop a behavioral plan for opposite action^j^, that allows cognitive disputation of thoughts and behaviors that perpetuate a negative emotional state
Ability to control impulsive behaviors and behave in accordance with desired goals when experiencing negative emotions	Decision making: (a) to remain focused on goals despite strong emotions; (b) to implement a considered plan to solve a problem; (c) to make decisions that are in line with how you want to feel	Switch on your decision making brain	Psychoeducation on exercising self-control in the pursuit of enduring values and goals^k^; delaying impulsive urges or responses to negative emotion; engaging in action based in order to achieve enduring valued goals^l^; a stop and observe skill; and a five step problem solving node-link exercise
Ability to control impulsive behaviors and behave in accordance with desired goals when experiencing negative emotions	Identity & values: (a) to know your personal values, goals and strengths; (b) to be aware of what motivates you; (c) to know who you are and how you want to live your life	No matter how you feel, do what matters to you	Psychoeducation of emotional avoidance as a maladaptive emotion regulation strategy; delivered through metaphor of a young person driving a minivan on a road trip; thoughts and feelings represented as passengers, goals as road signs, and values as a compass to represent actions in the pursuit of valued goals^m^.
		What do you stand for?	Psychoeducation on values^l^; values identification node-link mapping exercise.

a Nolen-Hoeksema and Watkins (2011)

b CBT functional analysis (Beck, 1976, 1995);

c Evolutionary model of emotions (Gilbert, 2009);

d Self-soothing (Linehan, 1993);

e Adaptation of white bear exercise (Wegner et al., 1987);

f Behavioural experiment (Bennett-Levy et al., 2004);

g Adapted from Unified Protocol (Barlow et al., 2011);

h Adapted from 3 min breathing space Mindfulness Based Cognitive Therapy exercise (Teasdale et al., 2014);

i Mindfulness definition adapted from Kabat-Zinn (2013);

j Adapted distress tolerance exercise, opposite action (Linehan, 2015);

k Duckworth et al. (2016);

l Adapted from values-based action from Acceptance and Commitment Therapy (Hayes et al., 1999, 2004);

m *Adapted from passengers on the bus (Hayes et al., 1999)*.

The processes and strategies within each domain are addressed through a series of exercises that are presented in worksheets and tools. Each of the 15 ERIC exercises delivered in the present pilot included the following elements: (a) psychoeducation delivered via a narrative, experiential exercise, or analogy; (b) a behavioral exercise involving node-link mapping (Dansereau and Simpson, 2009); and (c) a practice and reflection schedule to prompt daily repetition of new skills. For example, ERIC includes a grounding skill that progressively steps young people through sensory experiences in order to interrupt ruminative thinking. The concept of rumination is described in both a narrative form and the analogy of a “time traveling mind.” Through this analogy rumination is described as analogous to a time traveler who travels to the past and the future but does not spend time in the present. Through a progressive sensory process, young people then learn the skill to interrupt rumination by bringing the time traveler back to the present. The steps are represented in a node-link map for ease of understanding and recall. ERIC is a CBT informed program and includes many acceptance (i.e., non-avoidance of difficult emotions and thoughts) and mindfulness-based exercises, adapted for vulnerable young people. For example, ERIC includes brief mindfulness exercises such as a “Mindful Lean” which asks the young person to bring mindful attention to different parts of their body during the challenge of a physical grounding exercise delivered while leaning forward (i.e., feeling the toes gripping the ground to prevent falling). The ERIC exercises were adapted through a co-design process with vulnerable young people and were informed by contemporary evidenced-based manualized CBT interventions (see Table 1). Each ERIC exercise finishes with a practice and reflect schedule, outlined on all worksheets, for young people to engage in between session practice of new ERIC skills. The ERIC intervention was manualized for the present pilot (Hall and Simpson, 2018) and all worksheets and resources can be accessed online at eric.org.au.

A typical example of the delivery of ERIC across the 12 week intervention period in the current study would include the delivery of: two worksheets from the Emotional Literacy domain aimed at identifying emotions, recognizing their function and learning how emotions impact thoughts, behaviors and body signals (i.e., Dissecting Your Feelings: 5 Parts to Strong Emotions; Emotions are Our Mind's Inbuilt Alarm System); two worksheets from the Reducing Vulnerability domain aimed at reducing rumination and increasing skills in self-care (i.e., Five Self-care Habits; Interrupt Rumination with 5-4-3-2-1); one worksheet from the Tolerating Discomfort domain that taught young people to use actions to shift their attention from negative emotions (i.e., Shake Off Feelings); and finally one worksheet from the Identity and Values domain that identified personal values that assisted young people in acting in ways that help them pursue meaningful action in spite of strong emotions (i.e., No matter how you feel, do what matters to you). Table 1 provides further details of each of the exercises described above.

### Current Study

The primary aim of this one armed open pilot trial was to examine the impact of 12 weeks exposure to ERIC in combination with usual care on young peoples' abilities to regulate emotions. It was hypothesized that in comparison to baseline (Time 1), 6 weeks post the 12 week ERIC intervention period (Time 2) participants would report a reduction in overall emotion dysregulation. The secondary aim was to investigate the impact of ERIC in adjunct to usual care on young people's (1) psychological distress and symptoms of anxiety, depression and stress; (2) acceptance and non-avoidance of difficult thoughts and feelings; and (3) mindfulness skills. It was hypothesized that participants would report reductions in symptoms of psychological distress, anxiety, depression and stress and increases in acceptance and non-avoidance, and mindfulness from Time 1 to Time 2. The third aim of the study was to examine whether the magnitude of change in emotion regulation after the ERIC intervention moderated the magnitude of change in young people's symptoms of distress, anxiety, depression, stress, acceptance and non-avoidance, and mindfulness. It was predicted that change in emotion regulation would moderate the changes observed in psychological outcome variables, such that those who were found to have the greatest improvement in emotion regulation would also report the greatest improvements in distress, anxiety, depression, stress, acceptance and non-avoidance, and mindfulness.

## Methods

### Participants

Participants were young people recruited from community health centers, youth AOD services, youth primary mental health services, youth justice centers, and youth detention centers from metropolitan and regional Australia. Seventy-nine young people *(M* = 19.30 years; *SD* = 2.94) completed measures at Time 1 [40 males (50.6%), 37 females (46.8%), and two participants (2.5%) who identified as neither]. Most participants were born in Australia (92.4%), with ~20% identifying as Aboriginal and/or Torres Strait Islander. Most participants engaged with AOD or community services (51.9%), with fewer engaging with primary youth mental health services (32.9%) and justice or detention services (15.2%). Most participants had been receiving usual care with their current practitioner for at least 1 month prior to baseline (70.9%). The 6-week post-intervention follow-up was completed by 42 (53.2%) participants. Eligibility criteria included aged between 16 and 25 years and capable of giving informed consent. Exclusion criteria included active psychosis, an acute crisis presentation or active suicidality.

### Procedure

#### Study Design

This study was a single arm open trial comparing baseline (Time 1) to a six-week post-intervention follow up (Time 2) to examine the effect of 12 weeks of exposure to ERIC as an adjunct to usual care, on the primary outcome variable: (1) emotion regulation (DERS-Total). The impact of ERIC on the following secondary outcome variables was also examined (2) symptoms of anxiety (DASS-Anxiety); (3) symptoms of depression (DASS-Depression); (4) symptoms of stress (DASS-Stress); (5) symptoms of distress (DASS-Total); (6) acceptance and non-avoidance of difficult thoughts and feelings (AAQ-II); and (7) mindfulness-based skills (CAMS).

#### Study Protocol

The relevant university and hospital human ethics committees approved this study. The intervention was delivered over 12 weeks by frontline youth practitioners who were trained in ERIC and engaged in the coaching and fidelity checks outlined below. During the 12-week intervention period, practitioners delivered ERIC exercises and skills as an adjunct to usual care. Usual care was diverse across the settings and included outreach support, housing support, outpatient AOD counseling, case management, supervision of community-based orders, and offense-focused interventions. Six weeks following the completion of the 12-week intervention period Time 2 measures were administered and participants were reimbursed with a $20 gift card.

#### Practitioner Training and Intervention Fidelity

A series of 1-day ERIC workshops were held over the 2 year study period for the 21 practitioners, who included youth access workers, caseworkers, counselors, social workers, psychologists, and team leaders. The ERIC intervention modules and accompanying worksheets were delivered flexibly by practitioners based on the presentation of the participating client. During the 12-week intervention period, practitioners participated fortnightly in consultation and coaching sessions. Practitioners received performance feedback during these sessions to ensure fidelity to the intervention. Mid-way through the intervention period, practitioners submitted an audio-recorded role-play, allowing their coach to review their competence in the intervention delivery.

### Measures

#### Emotion Regulation

The 16-item version of the Difficulties in Emotional Regulation Scale (DERS-16; Bjureberg et al., 2017) assesses individuals' typical levels of emotional dysregulation. across five separate domains: non-acceptance of negative emotions; inability to engage in goal directed behaviors when experiencing negative emotions; difficulties controlling impulsive behaviors when experiencing negative emotions; limited access to emotion regulations strategies perceived as effective; and lack of emotional clarity. Items are scored on a 5-point Likert scale, with higher scores reflecting greater difficulty regulating emotions. The McDonald's omega reliability of the DERS-16 total score in the current study was high (Baseline, ω = 0.97; Followup, ω = 0.97).

#### Mental Health

The Depression, Anxiety and Stress Scales (DASS-21: Lovibond and Lovibond, 1995) contains seven items for each of the three scales. Participants rate the extent to which they experienced each symptom during the past week on a 4-point scale where higher scores indicate higher severity of symptoms of depression, anxiety, and stress, with higher scores on the total scale indicating greater psychological distress. Scores on the DASS-21 are doubled to align with scores on DASS-42 (Lovibond and Lovibond, 1995). Using McDonald's omega, the reliability was high for the DASS total (Baseline, ω = 0.92; Followup, ω = 0.94), depression (Baseline, ω = 0.88; Followup, ω = 0.92), anxiety (Baseline, ω = 0.80; Followup, ω = 0.84), and stress (Baseline, ω = 0.83; Followup, ω = 0.84) subscales.

#### Acceptance, Non-avoidance, and Psychological Flexibility

The Acceptance and Action Questionnaire Version 2 (AAQ-II; Bond et al., 2011) is a 7-item measure that assesses avoidance of difficult thoughts and emotions (experiential avoidance), and readiness to take action based on values. Items are scored on a 7-point scale with lower scores indicating greater levels of acceptance and non-avoidance of difficult thoughts and emotions. Using McDonald's omega, the reliability of the AAQ-II total score was high (Baseline, ω = 0.92; Followup, ω = 0.95).

#### Mindfulness

The Cognitive and Affective Mindfulness Scale (CAMS: Feldman et al., 2007) is a 12-item scale of mindfulness in general daily experiences which assesses the willingness and ability to be mindful. Items are rated on a 4-point scale with higher scores indicating greater mindfulness. Using McDonald's omega, the reliability of the CAMS total score was acceptable (Baseline, ω = 0.74; Followup, ω = 0.77).

### Statistical Analyses

Differences between completers and non-completers were tested using independent samples *t*-tests for continuous variables (age, and baseline scores on DERS-Total, DASS-Total, DASS subscales, AAQ-II and CAMS) and chi square tests for categorical variables (gender, service type and Aboriginal and Torres Strait Islander status). To account for the clustered nature of the data (i.e., timepoints nested within participants), we used linear mixed effects regression (with random intercept) to examine change over time across the primary outcome variable (i.e., DERS-Total) and secondary outcome variables (i.e., DASS total score and subscales, AAQ-II and CAMS). Specifically, we fit a series of models in which primary and secondary outcome variables were regressed onto time point (i.e., baseline vs. follow-up), and covariates of age, gender (male vs. female vs. other), service type (AOD and Community vs. Justice and Detention vs. Mental Health) and Aboriginal and/or Torres Strait Islander status (no vs. yes). Supplementary analyses (see Supplementary Table 1) found no evidence to suggest that a random (time) slope parameter was necessary to be included in any model. The magnitude of mean change over time was quantified using Cohen's d_av_, a measure of standardized mean change for correlated data (Lakens, 2013). A subsequent series of analyses examined whether changes in DERS-Total score from baseline to follow-up moderated changes in secondary outcome variables from baseline to follow-up. To do this, we first obtained standardized residuals from the univariable linear regression of DERS-Total score at follow-up on DERS-Total score at baseline. The standardized residuals from this model represent the magnitude of change in DERS-Total score over time, with positive scores indicating an increase in DERS-Total score over time. This residualized change score was then used as a moderator in the analyses specified above for change in secondary outcome variables over time. Moderation effects were explored through an examination of the relationship between the residualized change in DERS-Total, and the magnitude of the change in secondary outcome variables from baseline to follow-up. Mediation analyses were not estimated in the current study because of the strict assumptions underlying causal mediation analyses with only two time points (due to the lack of temporal dissociation; Cole and Maxwell, 2003), especially in the context of the low power of the sample size to detect mediation effects. For inferential analyses, missing data was accounted for using a full information maximum likelihood (FIML) approach to missingness (Kerkhoff and Nussbeck, 2019). All analyses retained the two participants who reported “Other” gender, but we also examined whether removing these participants influenced any results. The interpretation of all findings were robust (i.e., magnitude, direction, and *p*-values were consistent) to the removal of these participants.

## Results

### Clinical and Demographic Characteristics

Baseline characteristics were measured in relation to the 2 weeks prior to baseline data collection and are presented in Table 2.

**Table 2:** Baseline characteristics (*N* = 79).

**Characteristic**	***n***	**%^c^**
Unstable accommodation^a^ (*n* = 73)^b^	7	8.86
Work^d^ (*n* = 73)	22	27.85
Study^d^ (*n* = 73)	29	36.71
Illicit drugs exc. Cannabis^e^ (*n* = 79)	36	45.57
Alcohol^e^ (*n* = 79)	42	53.16
Cigarettes^e^ (*n* = 79)	53	67.09
Cannabis^e^ (*n* = 63)	28	35.44
Risky alcohol use^f^ (*n* = 79)	42	53.16

a Any days of unstable accommodation in 2 weeks prior to baseline;

b Number of participants with complete data on each characteristic;

c Proportions are of total sample.;

d Any days of engagement in 2 weeks prior to baseline;

e Any use of substance in 2 weeks prior to baseline;

f *measured using the AUDIT-C; Bradley et al., 2003. Both “Other” gender participants were classified as risky regardless of whether male or female cut-offs were applied*.

Mental health symptoms were examined through the DASS-42 clinical cut off scores (Lovibond and Lovibond, 1995). As indicated in Table 3, a substantial proportion of the participants scored in the severe-extremely severe range for symptoms of depression, anxiety and stress.

**Table 3:** Frequency and proportion of participants in each DASS-42 clinical cut-off category.

**Measure**	**DASS-depression^a^**	**DASS-anxiety^b^**	**DASS-stress^c^**
	**n (%)**	**n (%)**	**n (%)**
Normal	13 (16.46)	14 (17.72)	21 (26.58)
Mild-moderate	35 (44.30)	26 (32.91)	37 (46.84)
Severe-extreme	31 (39.24)	39 (49.37)	21 (26.58)

a DASS-Depression, Depression Anxiety and Stress Scale – Depression Scale;

b DASS-Anxiety, Depression Anxiety and Stress Scale – Anxiety Scale;

c *DASS-Stress, Depression Anxiety and Stress Scale – Stress Scale*.

### Attrition

Of the 79 participants recruited into the study, 42 (53%) completed both baseline and follow-up assessments. Table 4 compares those who dropped out of service contact and the study (i.e., Non-completers) to those who completed both baseline and follow-up assessment across a range of variables measured at baseline. There was little evidence for systematic differences between completers and non-completers across primary or secondary outcome variables. There was no relationship between dropout and age [*t*_(77)_ = −0.29, *p* = 0.775), gender ($\chi_{\left(1\right)}^{2}$ = 1.61, *p* = 0.205; note that “other” gender could not be included in this analysis due to low frequency] or Aboriginal and/or Torres Strait Islander [$\chi_{\left(1\right)}^{2}$ = 0.71, *p* = 0.398]. However, service type was related to dropout; individuals from AOD and community services had higher dropout rates [$\chi_{\left(2\right)}^{2}$ = 9.54, *p* = 0.008], with a larger proportion dropping out (26/41 = 63.4%) compared to those from mental health services (7/26 = 26.9%).

**Table 4:** Comparison of completers and non-completers across baseline measures of mental health and emotion regulation.

**Variable**	**Completers**	**Non-completers**	***t*-statistic (*df)***	***p***
	**(*n* = 42)**	**(*n* = 37)**		
	***M* (SD)**	***M* (SD)**		
DERS-total^a^	49.88 (16.20)	46.81 (18.93)	0.78 (77)	0.440
DASS-total^b^	53.67 (21.92)	53.86 (27.31)	−0.03 (77)	0.974
DASS-depression^c^	18.33 (9.54)	17.89 (11.25)	0.19 (77)	0.851
DASS-anxiety^d^	15.15 (8.26)	16.67 (9.25)	−0.77 (77)	0.444
DASS-stress^e^	20.19 (7.48)	19.30 (9.69)	0.46 (77)	0.646
AAQ-II^f^	29.29 (9.82)	27.51 (11.58)	0.74 (77)	0.464
CAMS^g^	26.76 (4.85)	26.86 (5.44)	−0.09 (77)	0.929

a DERS-Total, Difficulties in Emotion Regulation Scale;

b DASS-Total, Depression Anxiety and Stress Scale;

c DASS-Depression, Depression Anxiety and Stress Scale – Depression Scale;

d DASS-Anxiety, Depression Anxiety and Stress Scale – Anxiety Scale;

e DASS-Stress, Depression Anxiety and Stress Scale – Stress Scale;

f AAQ-II, Acceptance and Action Questionnaire;

g *CAMS, Cognitive and Affective Mindfulness Scale-Revised*.

### Intervention Outcomes

Table 5 presents a series of mixed effects regression models (one per outcome variable) used to estimate the mean change from baseline to follow-up across primary and secondary outcome variables, adjusting for age, gender, service type and Aboriginal and/or Torres Strait Islander status. Decreases in DERS-Total, DASS-Total, DASS-Depression, DASS-Anxiety, DASS-Stress, and AAQ-II were observed over time. Notably, the effect size for changes over time were of moderate magnitude for DERS-Total, DASS-Total, DASS-Depression, DASS-Anxiety, DASS-Stress, and AAQ-II (Cohen's d_av_s ranging from −0.26 to −0.53). No change was observed in CAMS across time points. Baseline correlations between intervention outcomes are presented in Supplementary Table 2.

**Table 5:** Pooled change in marginal means from baseline to follow-up across DERS, DASS, AAQ-II, and CAMS (*N* = 79)^a^.

**Variable**	**Baseline (T1) M(SD)**	**Follow up (T2) M(SD)**	**Change over time T1 vs. T2**	**Cohen's d_**av**_**
(a) DERS-Total^b^	48.44 (17.37)	38.20 (20.99)	Δ −10.24^***^ 95% CI (−14.41, −6.06)	−0.53
(b) DASS-Total^c^	53.75 (24.27)	42.66 (33.80)	Δ −11.10^**^ 95% CI (−18.21, −3.98)	−0.38
(i) DASS-Depression^d^	18.13 (10.25)	14.23 (15.28)	Δ −3.89^*^ 95% CI (−7.40, −0.39)	−0.31
(ii) DASS-Anxiety^e^	15.86 (8.66)	13.12 (11.18)	Δ −2.74^*^ 95% CI (−5.13,−0.36)	−0.28
(iii) DASS-Stress^f^	19.77 (8.49)	15.04 (10.84)	Δ −4.73^***^ 95% CI (−6.98, −2.48)	−0.49
(c) AAQ-II^g^	28.45 (10.58)	25.23 (14.50)	Δ −3.22^*^ 95% CI (−6.04,−0.40)	−0.26
(d) CAMS^h^	26.81 (5.07)	27.79 (6.79)	Δ 0.98 95% CI (−0.38, 2.33)	0.17

Δ = mean change,

* p < 0.05;

** p < 0.01;

*** p < 0.001; 95% CI = 95% Confidence interval.

a All analyses adjusted for age, gender, service type and Aboriginal and/or Torres Strait Islander status.;

b DERS-Total, Difficulties in Emotion Regulation Scale; DASS-Total, Depression Anxiety and Stress Scale;

d DASS-Depression, Depression Anxiety and Stress Scale – Depression Scale;

e DASS-Anxiety, Depression Anxiety and Stress Scale – Anxiety Scale;

f DASS-Stress, Depression Anxiety and Stress Scale – Stress Scale;

g *AAQ-II, Acceptance and Action Questionnaire; CAMS, Cognitive and Affective Mindfulness Scale-Revised*.

### Moderation of Primary Intervention Outcomes

**Figure 1:**
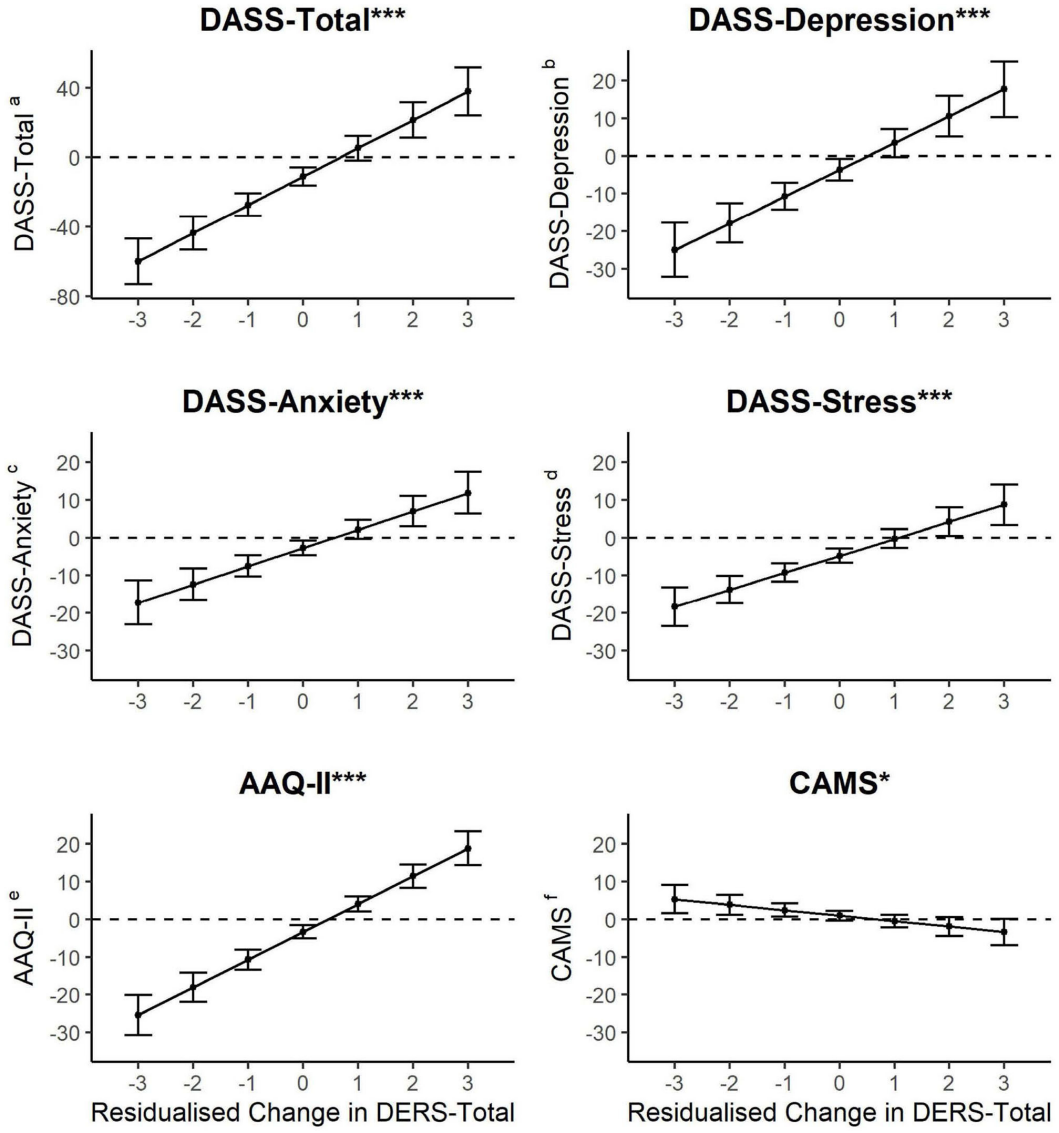
Relationship between residualised change in DERS-Total and magnitude of change in ^a^DASS-Total, ^b^DASS-Depression, ^c^DASS-Anxiety, and ^d^DASS-Stress, ^e^AAQ-II, and CAMS^f^ from baseline to follow-up. Note * moderation effect *p* < 0.05; *** moderation effect *p* < 0.001. Figures present effects and 95% confidence intervals. Confidence intervals that do not overlap zero represent statistically significant change from baseline to follow up.

Moderation analyses were conducted to examine whether each of the outcome effects over time (i.e., all those presented in Table 5) were influenced by the residualized change in DERS-Total score from baseline to follow-up. All analyses were adjusted for age, gender, service type and Aboriginal and/or Torres Strait Islander status. Figure 1 presents the change over time in all secondary outcome variables for different levels of residualized change in DERS-Total from baseline to follow-up. There was evidence that the residualized change in DERS-Total moderated the change over time in DASS-Total (*b* = 16.30, SE = 2.12, *p* < 0.001), DASS-Depression (*b* = 7.10, SE = 1.14, *p* < 0.001), DASS-Anxiety (*b* = 4.85, SE = 0.91, *p* < 0.001), DASS-Stress (*b* = 4.50, SE = 0.83, *p* < 0.001), AAQ (*b* = 7.38, SE = 0.78, *p* < 0.001), and CAMS (*b* = −1.44, SE = 0.57, *p* = 0.012). Inspection of Figure 1 reveals that individuals who had a low residualized change in DERS-Total (i.e., indicating improvement in DERS-Total after intervention) also had the greatest decrease (i.e., showing improvement) in DASS-Total, DASS-Depression, DASS-Anxiety, DASS-Stress, and AAQ-II, and greatest increase (i.e., showing improvement) in CAMS from baseline to follow-up. Contrastingly, there was evidence that individuals who worsened in DERS-Total (i.e., high residualized change score) also had worsening of DASS-Total, DASS-Depression, DASS-Anxiety, DASS-Stress, and AAQ-II from baseline to follow-up.

## Discussion

To our knowledge, this multi-sectoral pilot is the first to examine the impact of an adjunctive emotion regulation intervention on emotion regulation abilities in a cohort of vulnerable young people seeking treatment across youth AOD, community health, justice and mental health. Consistent with our predictions, the analyses revealed significant improvement in emotion dysregulation with a moderate effect size, 6 weeks after the 12-week exposure to ERIC, when delivered adjunctive to usual care. These findings are consistent with a previous study of the ERIC intervention with acutely vulnerable young people in residential drug rehabilitation (Sloan et al., 2018) and emotion regulation intervention studies in other cohorts of vulnerable young people including those with BPD and non-suicidal self-injury (NSSI; Bjureberg et al., 2017) and aggressive behaviors (Goldstein et al., 2018). The reduction in emotion dysregulation after exposure to ERIC is also consistent with findings from studies of other cognitive and behavioral interventions that target emotion regulation in adults with BPD and NSSI (Gratz et al., 2015) and Generalized Anxiety Disorder and Major Depressive Disorder (Mennin et al., 2018, Renna et al., 2017).

The secondary aim was to investigate the impact of exposure to ERIC alongside usual care on symptoms of anxiety and depression and the application of mindfulness and acceptance-based skills. A significant reduction in symptom severity of depression, anxiety and stress was observed 6 weeks after ERIC, in addition to a reduction in experiential avoidance and non-acceptance. A reduction in affective symptoms following an emotion regulation intervention is consistent with other studies (Renna et al., 2017) and would be expected given the nature of the intervention. Additionally, these findings are consistent with the literature on the impact of acceptance-based cognitive and behavioral interventions on emotion regulation and emotional disorders (Barlow et al., 2004). There was no main effect for change in mindfulness skills, which although inconsistent with other mindfulness-based intervention literature, may be indicative of the minimal “dose” of mindfulness in the ERIC intervention (i.e., two mindfulness exercises). Furthermore, adherence to self-directed regular practice of these two exercises in participants may have been low, suggesting regular and facilitated practice of mindfulness is probably required for this cohort. Nevertheless, as explained in later sections, there was evidence for improved mindfulness over time only in individuals who also reported an improvement in emotion regulation.

Finally, we examined whether the magnitude of change in emotion regulation after the ERIC intervention moderated the change in young people's symptoms of psychological distress, anxiety, depression, stress, mindfulness and acceptance-based skills. The examination of this moderation effect was of interest to test whether those participants whose emotion regulation improved between time points had the greatest improvements in each outcome over time. Indeed, for all outcomes, improvement was strongest for those participants whose emotion regulation also improved. Conversely, for participants whose emotion regulation became substantially worse, there was also evidence that their psychological distress (DASS-Total, depression, anxiety and stress) and avoidance of difficult thoughts and emotions increased (AAQ-II). Though the limitations of the present study design prevent definitive conclusions, the findings suggest that emotion regulation may be an important process influencing treatment outcomes in this cohort of vulnerable young people. Studies with repeated measurement of emotion regulation throughout treatment will help understand this relationship and provide greater evidence for causation. Nonetheless, this finding is consistent with the growing body of literature proposing that emotion regulation is a mechanism of change in treatment for emotional and behavioral disorders (for a review, see Sloan et al., 2017), with recent studies demonstrating that a change in emotion regulation difficulties mediates improvements in cognitive and affective features of BPD and NSSI in young people (Bjureberg et al., 2017); aggression, anxiety, and depressive outcomes in children (Burke and Loeber, 2016); BPD symptoms and deliberate self-harm in adult women (Gratz et al., 2015); attachment and interpersonal problem in adult survivors of childhood maltreatment (Keating et al., 2018); and PTSD symptoms and conduct problems in children and adolescents (Sharma-Patel and Brown, 2016). Taken together, the findings from the present study and the current literature suggest emotion dysregulation is an important treatment target across the range of psychopathological symptomatology relevant to vulnerable young people. However, well-controlled studies are needed to provide stronger evidence that emotion regulation is the mechanism of change in the transdiagnostic treatment of complex presentations, including substance related issues, antisocial behaviors, self-harm and emotional and affective disturbance.

### Strengths and Limitations

A strength of this study was that participants were highly heterogeneous and were recruited from a range of youth services thus enhancing the ecological validity of the outcomes of this study. The sample consisted of vulnerable young people (20% identified as Aboriginal and/or Torres Strait Islander) with polysubstance use, harmful alcohol use, high levels of mental health issues, criminal justice system involvement and housing difficulties. Their level of complexity and vulnerability is consistent with previous service delivery studies undertaken in youth AOD populations both within Australia (Mitchell et al., 2016) and internationally (James et al., 2013). It is likely that the complexity of the sample contributed to the high rates of attrition seen in the current study. Unlike the large majority of clinical trials in young people which are consistently criticized for their lack of generalizability to treatment seeking populations (Weisz et al., 2017) this study was undertaken in partnership with clinical services and was arguably more representative of actual service users. Indeed, the attrition rates in the current study were comparable to other studies of treatment seekers with polysubstance use (e.g., Hall et al., 2018) and psychosocial complexity (Ferguson et al., 2020). Nonetheless, this limitation is somewhat ameliorated by statistical analyses adopted which takes into account missing data and the fact that completers and non-completers were found to report no baseline differences in basic demographics and severity of emotion dysregulation or mental health symptoms. Interestingly, those attending AOD or community services were more likely disengage than those in mental health services. Finally, given that this is an open pilot study, with no control condition, the results must be interpreted with caution until a randomized control trial can be conducted that includes a matched comparison study of service users who receive treatment as usual only. This will then able us to examine the additional benefits of ERIC over usual care, which is of particular interest given our findings that emotion regulation was a potential mechanism of action for a range of reductions in mental health symptoms in this study.

## Conclusion

Emotion regulation based cognitive and behavioral therapies represent a promising avenue for improving the care available to vulnerable young people with coexisting and complex transdiagnostic presentations. The ERIC intervention was co-designed and piloted in young people with complex psychosocial presentations and with high frequencies of severe psychological distress. The reductions in the severity of emotion dysregulation and anxiety are promising and were evident despite the complexity of the participants and the diversity of the service setting. In the present study, ERIC was delivered by youth practitioners alongside usual care, highlighting the translational nature of this approach, thereby having promise in helping to address the science-to-service gap commonly encountered within youth services. Finally, improvement across secondary outcomes was observed only for those participants whose emotion regulation also improved, adding credence to the notion that emotion regulation is an underlying mechanism that can be targeted within treatment within this cohort.

## Data Availability Statement

The raw data supporting the conclusions of this article will be made available by the authors, without undue reservation.

## Ethics Statement

The studies involving human participants were reviewed and approved by Deakin University Human Research Ethics Comittee and the relevant Hospital Ethics Committees. Written informed consent to participate in this study was provided by participants. If participants were unable to provide consent due to age, a legal guardian or parent provided informed consent.

## Author Contributions

All authors listed have made a substantial, direct and intellectual contribution to the work, and approved it for publication.

## Conflict of Interest

The authors declare that the research was conducted in the absence of any commercial or financial relationships that could be construed as a potential conflict of interest.
