# Supplementary material for: An Emotion Regulation and Impulse Control (ERIC) Intervention for Vulnerable Young People: A Multi-Sectoral Pilot Study
Source: Front Psychol. 2021 Apr 1;12:554100. doi: 10.3389/fpsyg.2021.554100 (PMC8047628; doi:10.3389/fpsyg.2021.554100)
Supplement: Supplementary file 1 [file Table_1.pdf]

Supplementary Table 1: *Likelihood Ratio Tests Comparing Random Intercept Only Models to Random Intercept + Random (Time) Slope Models*

	Random intercept only				*Random intercept + random (time) slope*				
	LL	df	AIC	BIC	LL	df	AIC	BIC	Likelihood Ratio Test
DERS-Total	-485.312	9	988.6238	1013.636	-485.312	10	990.6238	1018.415	LR chi2(1) < 0.01; p = 1.00
DASS-Total	-531.292	9	1080.585	1105.597	-531.198	10	1082.396	1110.187	LR chi2(1) = 0.19; p = .664
DASS-Depression	-434.411	9	886.8216	911.8337	-434.198	10	888.3967	916.1879	LR chi2(1) = 0.42; p = .515
DASS-Anxiety	-406.229	9	830.4584	855.4705	-406.229	10	832.4584	860.2496	LR chi2(1) < 0.01; p = 1.00
DASS-Stress	-405.591	9	829.1814	854.1935	-405.591	10	831.1814	858.9727	LR chi2(1) < 0.01; p = 1.00
AAQ-II	-427.777	9	873.5538	898.5659	-427.737	10	875.4732	903.2644	LR chi2(1) = 0.08; p = .776
CAMS	-349.704	9	717.4071	742.4192	-349.704	10	719.4071	747.1983	LR chi2(1) < 0.01; p = 1.00

LL = Loglikelihood; AIC = Akaike information criterion; BIC = Bayesian information criterion; df = degrees of freedom; LR chi2 = Likelihood Ratio chi2; DERS-Total = Difficulties in Emotion Regulation Scale; DASS-Total = Depression Anxiety and Stress Scale; DASS-Depression = Depression Anxiety and Stress Scale – Depression Scale; DASS-Anxiety = Depression Anxiety and Stress Scale – Anxiety Scale; DASS-Stress = Depression Anxiety and Stress Scale – Stress Scale; AAQ-II = Acceptance and Action Questionnaire; CAMS = Cognitive and Affective Mindfulness Scale-Revised
