# Supplementary material for: An Emotion Regulation and Impulse Control (ERIC) Intervention for Vulnerable Young People: A Multi-Sectoral Pilot Study
Source: Front Psychol. 2021 Apr 1;12:554100. doi: 10.3389/fpsyg.2021.554100 (PMC8047628; doi:10.3389/fpsyg.2021.554100)
Supplement: Supplementary file 2 [file Table_2.pdf]

Supplementary Table 2: ***Baseline correlations of the clinical measures***

	**DERS-Total[a]**	**DASS-Total[b]**	**DASS-Depression[c]**	**DASS-Anxiety[d]**	**DASS-Stress[e]**	**AAQ-II[f]**
**DERS-Total**						
**DASS-Total**	.76***					
**DASS-Depression**	.72***	.88***				
**DASS-Anxiety**	.60***	.88***	.62***			
**DASS-Stress**	.69***	.90***	.67***	.75***		
**AAQ-II**	-.53***	-.51***	-.44***	-.48***	-.44***	
**CAMS[g]**	.79***	.86***	.81***	.74***	.72***	-.51***

Note. ***p<.001, **p<.01, *p<.05
[a]DERS-Total, Difficulties in Emotion Regulation Scale ; [b]DASS-Total, Depression Anxiety and Stress Scale [c]DASS-Depression, Depression Anxiety and Stress Scale – Depression Scale ; [d]DASS-Anxiety, Depression Anxiety and Stress Scale – Anxiety Scale ; [e]DASS-Stress, Depression Anxiety and Stress Scale – Stress Scale; [f]AAQ-II, Acceptance and Action Questionnaire; [g]CAMS, Cognitive and Affective Mindfulness Scale-Revised
